# Inferior Phrenic Artery Variations and a Novel Classification of Aortic and Non-aortic Origins: An Observational CT Angiographic Study

**DOI:** 10.7759/cureus.84960

**Published:** 2025-05-28

**Authors:** Garima Sehgal, Anit Parihar, Nikhil Aggarwal, Mariam Moonis

**Affiliations:** 1 Anatomy, King George's Medical University, Lucknow, IND; 2 Radiodiagnosis, King George's Medical University, Lucknow, IND; 3 Anatomy, Army College of Medical Sciences, New Delhi, IND; 4 Anatomy, Dr. Ram Manohar Lohia Institute of Medical Sciences, Lucknow, IND

**Keywords:** angiography, aorta, displaced, hemoptysis, hepatocellular carcinoma, variations

## Abstract

Background and aim

The inferior phrenic arteries (IPAs) usually originate from the abdominal aorta as collateral branches and are small arteries that supply the diaphragm. Related pathologies include hemoptysis, bleeding during surgery or trauma, Mallory-Weiss tear, gastroesophageal cancer, and collateral blood supply to hepatocellular carcinoma. Knowledge about variations involving these arteries is important for clinicians, radiologists, and surgeons. This study aims to observe variations in the origin of IPAs and propose a novel classification of aortic and non-aortic origin arteries based on CT angiographic findings.

Materials and methods

The study was conducted in the Department of Anatomy in collaboration with the Department of Radiodiagnosis. Computerized tomographic angiograms of 100 subjects aged 15-70 years were retrospectively collected and reviewed after applying inclusion and exclusion criteria. The site and pattern of origin of the IPAs were observed on reconstructed CT images. The arteries were grouped according to the type of variation observed, and the prevalence of specific variations was estimated and compared on the basis of gender. A novel classification of arterial variants was also proposed.

Results

IPA anatomy was studied in 100 subjects. Instead of the expected 200 IPAs, 203 arteries were observed due to the presence of three accessory arteries. Out of these 203 arteries, 131 (64.53%) were of aortic origin - 113 (86.26%) with normal origin and 18 (13.74%) with variant (displaced) origin. The remaining 72 arteries (35.47%) were of non-aortic origin (misplaced arteries). Thus, two types of arterial variations were identified: displaced arteries (variant aortic origin) and misplaced arteries (non-aortic origin).

Conclusions

The total prevalence of variations in IPAs was found to be 44.34%. Precise knowledge of these variations is crucial for medical management and before surgical or interventional treatments. The study uniquely reports the displaced variants of IPAs and proposes a classification into different types based on the pattern of origin.

## Introduction

The inferior phrenic arteries (IPAs) usually arise from the abdominal aorta as collateral branches and are small-caliber arteries responsible for supplying the diaphragm [1]. They typically originate superior to the celiac trunk at the level of the 12th thoracic vertebra [2]. Apart from the diaphragm, the IPAs also supply several adjacent structures, including the adrenal glands, esophagus, stomach, liver, inferior vena cava (IVC), and retroperitoneum. The IPAs thus play a critical role in maintaining the vascular integrity of both thoracic and upper abdominal organs.

The right IPA (RIPA) and left IPA (LIPA) have recently gained prominence due to their recognition as important sources of extrahepatic collateral arterial pathways supplying hepatic malignancies, particularly hepatocellular carcinoma (HCC). This collateral circulation is vital in cases of hepatic artery occlusion or after repeated chemoembolization procedures [3].

Following their origin from the aorta, the IPAs ascend laterally, traversing in front of the crura of the diaphragm, medial to the suprarenal glands. The LIPA passes posterior to the esophagus, whereas the RIPA passes posterior to the IVC, subsequently proceeding toward their respective sides [4]. Close to the central tendon of the diaphragm, both arteries typically bifurcate into anterior and posterior branches. Additionally, anatomical studies have highlighted their contribution to suprarenal and superior adrenal branches, indicating their relevance in adrenal surgeries and pathologies.

Numerous variations in the origin of the IPAs have been reported, including origins from the celiac axis (either as a common trunk or independently), renal arteries, left gastric artery, hepatic artery, superior mesenteric artery (SMA), and gonadal arteries, as well as the presence of accessory vessels [3,5]. Variations in the IPA origin may complicate surgical procedures such as diaphragmatic repairs, adrenalectomy, upper gastrointestinal surgeries, or interventions targeting hepatic malignancies.

During the course of an initial study on the SMA and inferior mesenteric artery, anomalous origins of the IPAs were frequently encountered, prompting a detailed analysis specifically focused on the IPAs.

Given the frequent and clinically significant variations in the topographic anatomy of the IPAs, the present study was undertaken to investigate variations in the site and level of origin of this surgically important artery. Precise knowledge of such variations is essential for optimizing the clinical, radiological, and surgical evaluation of conditions where the IPAs are involved. Prior identification of atypical origins becomes especially crucial in planning interventional radiological procedures, such as transcatheter arterial chemoembolization (TACE), and in minimizing the risk of inadvertent injury during upper abdominal surgeries. Moreover, awareness of anomalous IPA patterns can aid in the diagnosis and management of upper gastrointestinal bleeding, adrenal tumors, and thoracoabdominal trauma.

This study aimed to analyze the anatomical variations in the origin of the IPAs using computed tomographic angiography (CTA). Specifically, it aimed to classify these variations based on their aortic or non-aortic origin and to propose a novel classification system for displaced aortic variants, with the goal of improving anatomical understanding relevant to surgical and interventional procedures.

## Materials and methods

This observational retrospective study was conducted from October 1, 2021 to May 1, 2022 in the Department of Anatomy, in collaboration with the Department of Radiodiagnosis, King George’s Medical University, Lucknow, India. Institutional Ethical Committee approval was obtained prior to the commencement of the study (approval IV PGTSC-IIA/P47). All procedures were carried out in accordance with the ethical standards of the institutional and/or national research committee and with the 1964 Helsinki Declaration and its later amendments.

CT angiography was performed using a 64-slice multidetector CT scanner (Philips Brilliance, Philips Healthcare, Amsterdam, Netherlands), employing standard contrast-enhanced abdominal imaging protocols. Scanning parameters included collimation of 0.625 mm, tube voltage of 120 kVp, tube current modulation with an effective mAs of 250-300, and slice thickness of 1 mm. A non-ionic contrast agent (100 mL, injected at 4.0 mL/sec) was used with the bolus tracking technique initiated at 150 HU in the abdominal aorta, and arterial phase imaging was acquired at a delay of approximately 18-22 seconds. Multiplanar and volume-rendered reconstructions were performed for optimal vascular delineation.

Retrospective data from 100 subjects aged between 15 and 70 years were collected and analyzed. The sample size was determined based on feasibility and availability of eligible CT angiography cases that met the inclusion criteria within the defined study period. Although formal statistical sample size calculation was not performed due to the retrospective nature of the study, similar previous CT-based anatomical studies have reported sample sizes ranging from 50 to 150 subjects. Hence, a sample size of 100 was considered adequate to observe anatomical variations in IPA origin and propose a meaningful classification framework.

Study subjects had undergone CT angiography of the abdomen as a workup for various medical or surgical indications unrelated to hepatic, diaphragmatic, or adrenal pathology. Inclusion criteria comprised radiologically normal abdominal scans without any structural anomaly, tumor, or growth affecting the region of interest. Exclusion criteria included images with evident pathological changes, previous surgical interventions, or poor image quality in the upper abdominal region (specifically involving the diaphragm, adrenal glands, and adjacent vascular structures) that precluded adequate visualization of the IPAs.

The axial source data, along with reformatted multiplanar reconstruction images and volume-rendered 3D images, were utilized to study the IPAs. Observations primarily focused on the origin, course, and branching patterns of the RIPA and LIPA.

The IPA was identified on CT scans as an artery ascending vertically through the abundant retroperitoneal fat overlying the diaphragmatic crura. The site of origin and the parent artery were meticulously observed and documented.

Based on the pattern of origin, the IPAs were categorized as normal or variant arteries. Arteries originating directly from the abdominal aorta at or above the level of the celiac axis - typically around the T12 vertebral level and after the aorta has traversed the diaphragm - were classified as arteries with normal aortic origin (normal IPAs). Any deviation from this standard pattern - whether a non-aortic origin or an altered aortic level - was categorized as a variant artery.

The prevalence of variations was recorded and further analyzed on the basis of gender. A novel classification system was also proposed to categorize aortic and non-aortic variants of IPAs based on their site and pattern of origin, including information about displaced aortic origins relative to key abdominal vascular landmarks (celiac trunk and SMA). This structured approach provides a clearer anatomical rationale for grouping aberrations from the normal pattern and offers potential clinical relevance for radiological and surgical interpretation.

## Results

The origin of IPAs was studied in 100 subjects, comprising 53 males and 47 females. Among these, variations in the origin of IPAs were observed in 52 (52%) of the subjects. Prevalence of variation among males (28; 52.83%) was marginally higher compared to females (24; 51.06%). However, no statistically significant difference was observed between the two genders with respect to the proportion of variants (p = 0.860). Table 1 shows the prevalence of normal and variant origins of the IPAs categorized according to gender.

**Table 1:** Prevalence of normal or variant IPA IPA, inferior phrenic artery

Gender	Subjects (n = 100)	Type of origin			
		Normal	% Normal	Variant	% Variant
Males	53	25 cases	47.17%	28 cases	52.83%
Females	47	23 cases	48.94%	24 cases	51.06%
Total	100	48	48%	52	52%

In total, 203 IPAs were identified in 100 subjects (one right and one LIPA in each subject, along with three accessory IPAs). Out of these, 131 arteries (64.53%) were of aortic origin, and 72 arteries (35.47%) were of non-aortic origin. Table 2 presents the distribution of aortic and non-aortic IPAs across genders.

**Table 2:** Distribution of aortic and non-aortic IPAs ^*^ due to additional accessory arteries CA, celiac artery; IPA, inferior phrenic artery; LGA, left gastric artery; LHA, left hepatic artery; RA, renal artery; SA, splenic artery

Gender	Subjects (n = 100)	Total arteries	Aortic IPAs	Non-aortic IPAs (CA/LGA/SA/LHA/RA)
Males	53	107^*^	67	40
Females	47	96^*^	64	32
Total	100	203	131	72

**Figure 1:**
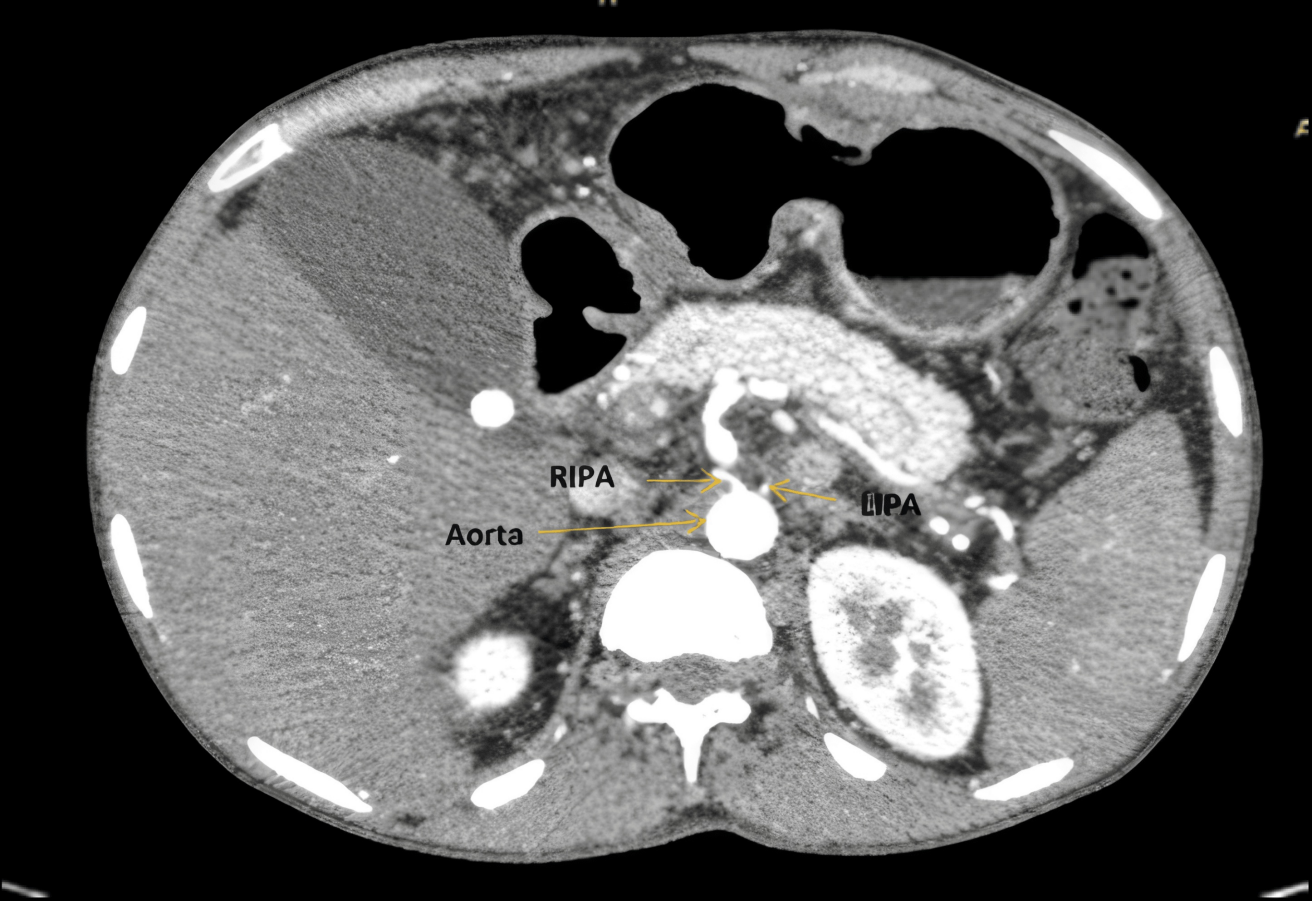
Axial CT image showing bilateral normal origin of aortic IPAs IPA, inferior phrenic artery; LIPA, left inferior phrenic artery; RIPA, right inferior phrenic artery

Among the total 203 arteries studied, a majority displayed normal aortic origin. To illustrate the typical normal anatomical course of the IPAs, an axial CT angiographic image depicting bilateral normal aortic origin was selected. This image confirms that both the RIPA and the LIPA arise symmetrically from the abdominal aorta without any displacement or abnormal branching pattern, as shown in Figure 1.

Among the total 203 IPAs observed, 72 (35.47%) arteries displayed a non-aortic origin. These non-aortic variant IPAs most commonly originate from the celiac artery, followed by the left gastric artery, renal artery, splenic artery, and left hepatic artery in decreasing order of frequency. It was observed that the celiac artery, left gastric artery, and splenic artery gave origin to both RIPA and LIPA, whereas the left hepatic artery gave origin only to LIPA, and the right renal artery only to RIPA.

Regarding the aortic origins, the localization of the site of origin was further analyzed. Among the 131 IPAs that originated from the aorta, 113 arteries (86.26%) exhibited normal origin, arising at or just above the level of the celiac axis, typically corresponding to the T12 vertebral level. In contrast, 18 arteries (13.74%) showed a displaced aortic origin, emerging at levels caudal to the celiac trunk, including between the celiac axis and the SMA (around L1), at the level of the SMA (L1), or even below it, approaching the level of the renal arteries (L1-L2).

The displaced aortic origins were further categorized based on their anatomical relationship with major ventral branches of the abdominal aorta, using these as reference landmarks. Specifically, 10 arteries (7.63%) originated below the celiac trunk and above the SMA - a region typically corresponding to the T12-L1 vertebral level. Two arteries (1.53%) were observed to arise at the level of the SMA, approximately L1, while six arteries (4.58%) originated below the SMA, approaching the L1-L2 level, near the origin of the renal arteries. This stratified assessment allowed for clearer anatomical localization of displaced IPA origins. Representative CT angiographic images were selected to demonstrate these levels and variations.

**Figure 2:**
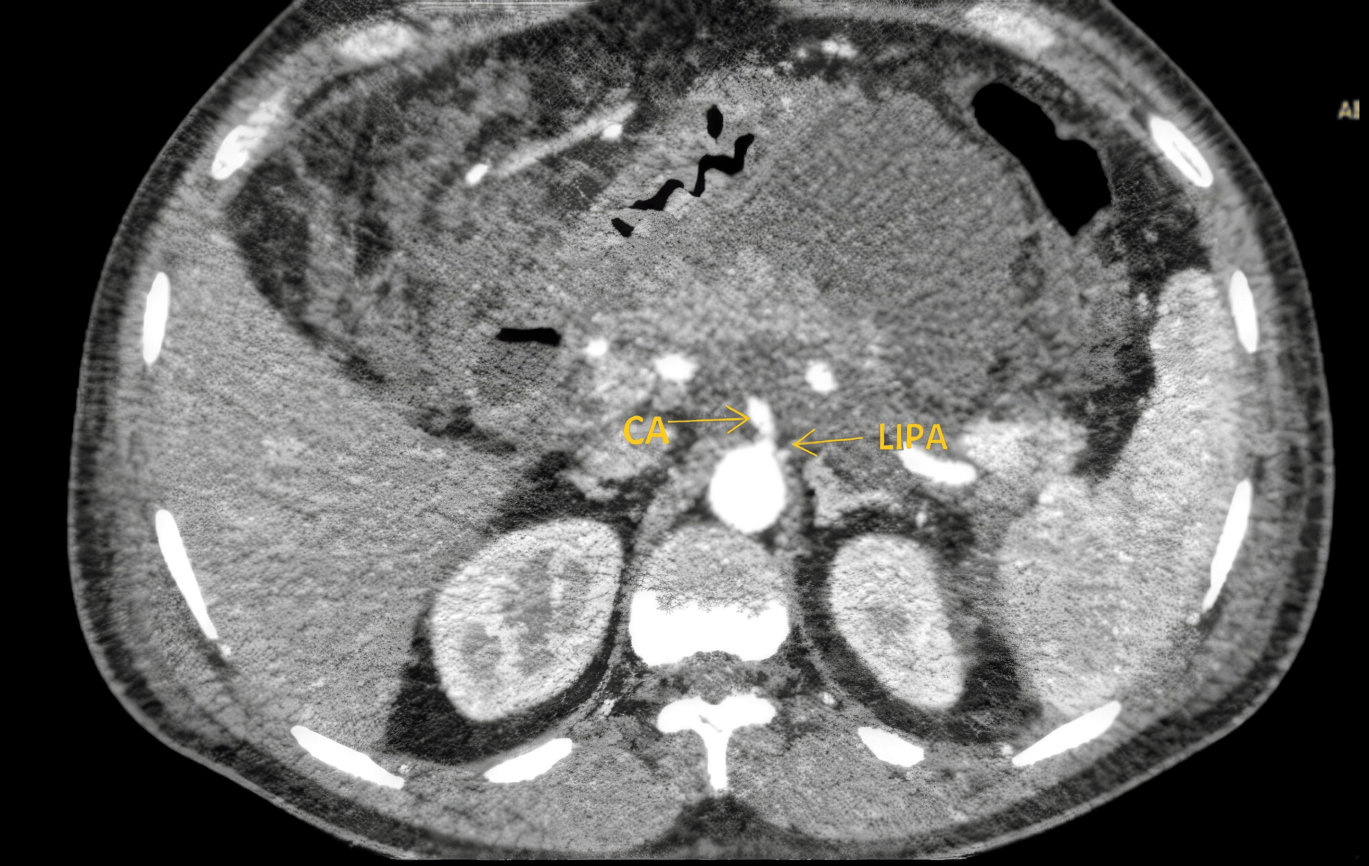
Axial CT image showing the origin of LIPA from the aorta below the level of the CA and above the SMA CA, celiac artery; LIPA, left inferior phrenic artery; SMA, superior mesenteric artery

An example of a LIPA originating from the abdominal aorta below the level of the celiac artery and above the SMA is illustrated in Figure 2. This image highlights the anatomical displacement of the LIPA origin relative to standard vascular landmarks.

**Figure 3:**
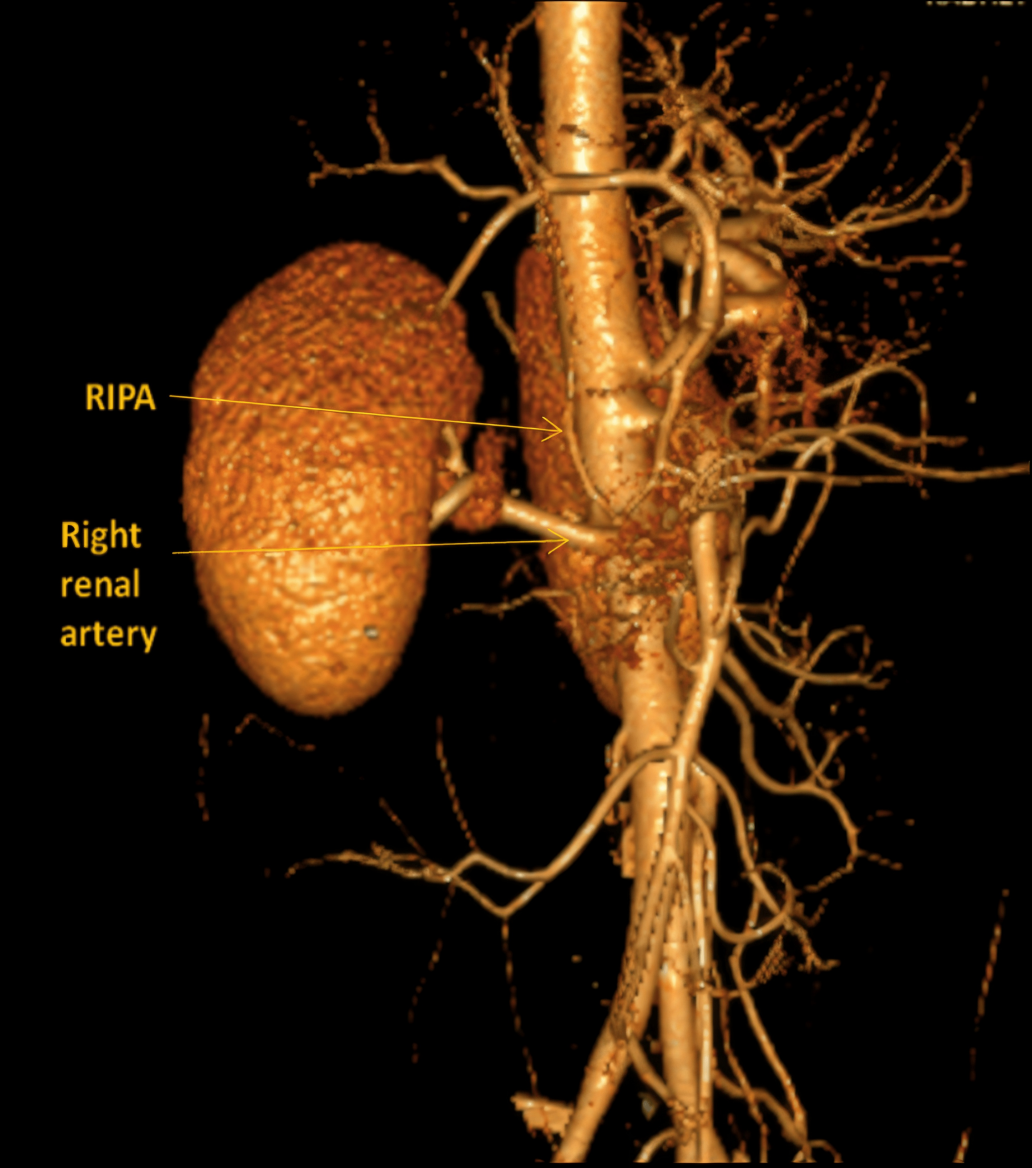
Volume-rendered CT image showing origin of RIPA from the aorta below the origin of the SMA RIPA, right inferior phrenic artery; SMA, superior mesenteric artery

Similarly, an instance of a RIPA originating from the abdominal aorta below the level of the SMA (approximately at the L1-L2 vertebral level), near the origin of the right renal artery, is depicted in Figure 3. This variation further emphasizes the extent of caudal displacement that can be encountered in clinical practice.

Upon comparing the side-specific variations, the LIPA exhibited more frequent anomalies - LIPA: 62 out of 203 (30.77%) vs. RIPA: 47 out of 203 (23.15%). Taking into account the site of origin of the LIPA and RIPA in each subject, the following distribution patterns were observed: independent origin of both RIPA and LIPA from the aorta was seen in 42 cases, a common trunk origin of RIPA and LIPA from the aorta was noted in six cases, only LIPA arising from the aorta with RIPA from a non-aortic source was found in four cases, only RIPA from the aorta with LIPA from a non-aortic source in three cases, and separate non-aortic origins of both arteries in 45 cases. Additionally, three accessory or supplemental arteries were identified, all on the left side. Among females, aortic-origin IPAs were more common, with 64 out of 96 arteries accounting for 66.67%. In males, 67 out of 107 arteries originated from the aorta, comprising 62.62%. Variant non-aortic IPAs were observed more frequently in males, with 23 LIPAs and 17 RIPAs, giving a total of 40 (37.38%) variant arteries. In females, 17 LIPAs and 15 RIPAs were observed, totaling 32 (33.33%) variant arteries. However, no significant difference was observed between the two genders with respect to the proportion of variants (p = 0.860).

## Discussion

The LIPA and RIPA are branches of the abdominal aorta that supply multiple vital structures, including the diaphragm, esophagus, stomach, liver, and adrenal glands. Anatomically, the right and left arteries are closely related to the IVC and the esophagus, respectively. Each artery gives off anterior, posterior, and suprarenal branches. The anterior branch of the RIPA supplies the diaphragm and IVC and additionally establishes vascular contact with the liver, especially at the bare area involving liver segments I, II, and VII. The anterior branches of the LIPA, in contrast, supply the left hemidiaphragm, esophagus, stomach, and spleen via accessory branches [6,7].

The posterior branches of the IPAs course laterally and anastomose with the lower posterior intercostal arteries and musculophrenic artery, forming important collateral pathways. Additionally, the IPAs may communicate with the internal mammary artery and the pericardiophrenic artery, highlighting their relevance during both thoracoabdominal and cardiothoracic surgeries [8,9].

The anatomical variations observed in the origins of the IPAs in our study can be attributed to the complex embryological development of the abdominal arterial system. During embryogenesis, the dorsal aorta gives rise to several paired lateral splanchnic branches that supply the developing suprarenal glands, gonads, and mesonephros. The IPAs are believed to originate from these lateral branches. Aberrations occur due to the persistence or regression of these embryonic vessels, leading to variations in the origin of the IPAs from the abdominal aorta, celiac trunk, renal arteries, or other nearby vessels. For instance, if a normally regressing lateral splanchnic branch persists, it may give rise to an IPA from an atypical source such as the renal artery. Conversely, regression of a branch that typically persists may result in altered or absent IPAs. These findings are consistent with previous studies describing similar arterial deviations in relation to developmental remodeling of the aortic branches [3,10,11]. Understanding these embryological mechanisms provides a developmental rationale for the classification proposed in our study and highlights the need for preoperative vascular mapping in hepatobiliary and adrenal interventions.

Kahn emphasized that the IPAs contribute significantly to the vascularization of the adrenal glands, underscoring the importance of angiographic evaluation during workup of adrenal pathologies. Variations in the anatomy of IPAs may therefore have direct implications for adrenal tumor embolization procedures [12].

Cadaveric studies evaluating IPA origin have reported significant variation. Pulakunta et al. observed in a study on 32 South Indian cadavers that 87.5% of IPAs originated from the aorta, 6.25% from the celiac trunk, 3.125% from the renal artery, and another 3.125% from other sources, including the left gastric, hepatic, and superior mesenteric arteries [13]. Similarly, Piao et al., in a study of 68 cadavers, found that IPAs originated from the aorta in 61.6% of cases, from the celiac trunk in 28.2%, and from either renal, left gastric, or middle adrenal arteries in 10.2% [14]. Pick and Anson also reported comparable findings, noting aortic (45.1%) and celiac trunk (47.8%) origins as predominant, with renal (5.8%), left gastric (2.3%), and hepatic (0.3%) origins being less frequent [1,2].

In a CT-based study, Gokan et al. reported that the aorta and celiac trunk were the most common sources for the IPAs, with the aortic origin being more frequent for the RIPA and the celiac origin more common for the LIPA. Alternative origins from the left gastric, hepatic, superior mesenteric, and spermatic arteries occurred in fewer than 4% of cases. Additionally, a notable 9% incidence of RIPA origin from the right renal artery was reported [15].

The objective of our study was to specifically analyze the site and source artery of IPA origin using CTA. In the present study, the majority of IPAs (64.53%) originated from the aorta, while 35.47% arose from non-aortic sources. These findings align with previous observations that have consistently identified the aorta and celiac trunk as the most common origins for IPAs.

Our study observed that variations in IPA origin were slightly more common in males (52.83%) than in females (51.06%), although this difference was not statistically significant (p = 0.860) (Table 1). These observations reaffirm the view that gender differences in IPA variation patterns are minimal.

Among the non-aortic origins, the celiac trunk was the most frequent source, followed by the left gastric artery, renal artery, splenic artery, and left hepatic artery. The celiac trunk, left gastric artery, and splenic artery were found to give rise to both RIPA and LIPA, whereas the left hepatic artery contributed only to LIPA and the right renal artery to RIPA. This is consistent with previous studies such as those by Pick and Anson and Gokan et al., wherein the celiac trunk was identified as a significant alternative source [1,2,15].

However, it is noteworthy that the percentage of variant IPAs arising from the celiac trunk in our study is lower than that reported in earlier studies, possibly due to differences in imaging modality used, population demographics, or subject selection criteria.

The RIPA has been described as a major collateral supply route for HCC, especially involving the caudate lobe and posterior segments of the liver [16]. Therefore, understanding its anatomical variations is critical for planning interventions such as TACE. In the present study, the RIPA originated from the aorta in 68% of cases, from the celiac trunk in 26%, from the right renal artery in 5%, and from the left gastric artery in 1%. Comparatively, the LIPA originated from the aorta in 61.17%, the celiac trunk in 31.07%, the left gastric artery in 4.85%, and the splenic or left hepatic artery in 2.91% of cases.

Kimura et al., studying 178 HCC patients undergoing chemoembolization, reported the RIPA origin as aorta (57%), celiac artery (30%), left gastric artery (2%), dorsal pancreatic artery (1%), and right renal artery (11%) [17]. Thus, the findings of our study reflect a higher prevalence of aortic-origin RIPA than previously reported.

Of particular interest is the high proportion of anomalous LIPA origins from the left gastric artery (4.85%) in our study. Previous studies, including those by Tanaka et al. and Ozbülbül et al., have reported a lower incidence of such findings [18,19]. Tanaka et al. noted a 1.7% origin of LIPA from the left hepatic or left gastric artery [18], whereas Ozbülbül et al. did not report any such anomaly, but they described LIPA arising from RIPA in 3.23% of cases, which was not observed in our study cohort [19].

Accessory LIPAs were identified in one male and two female subjects. Accessory arteries are clinically significant as they may act as additional feeders in cases of tumor vascularization or surgical bleeding. Cases of double IPAs supplying the lesser curvature of the stomach and duodenum have also been described in the literature [20,21].

The LIPA, owing to its contribution to the gastroesophageal junction vasculature, must be carefully considered in cases of upper gastrointestinal bleeding and in planning for gastrectomy and hiatal hernia surgeries [22]. Injuries to variant LIPA originating from the left gastric artery can result in intra-abdominal bleeding during surgical interventions. Repeated TACE procedures often result in stenosis of hepatic arteries; in such cases, hepatic perfusion is commonly reconstituted through collateral pathways, including the RIPA [18]. Understanding these collateral pathways is crucial to avoid inadvertent complications such as pleural effusion, diaphragmatic weakness, abdominal pain, and gastric or esophageal ulceration secondary to ischemia [19].

A comparison of the percentage prevalence of RIPA and LIPA origins between the present study and previous literature is summarized in Table 3.

**Table 3:** Comparison of percentage prevalence of RIPA and LIPA origins between the present study and other reported studies Ao, aorta; CA, celiac artery; DSA, digital subtraction angiography; HCC, hepatocellular carcinoma; LGA, left gastric artery; LHA, left hepatic artery; LIPA, left inferior phrenic artery; LRA, left renal artery; RIPA, right inferior phrenic artery; RRA, right renal artery; SA, splenic artery

Source artery	Gokan et al. (2001) [15] (CT study; 16 HCC cases)		Loukas et al. (2005) [6] (cadaveric; 200 subjects)		So et al. (2009) [23] (DSA and CT; 580 subjects)		Ozbülbül et al. (2011) [19] (cadaveric; 93 subjects)		Present study (CT study; 100 subjects)	
	RIPA	LIPA	RIPA	LIPA	RIPA	LIPA	RIPA	LIPA	RIPA	LIPA
Ao	31.25%	31.25%	38%	45%	57.30%	-	25.81%	22.58%	68%	61.17%
CA	37.50%	50%	40%	47%	29.31%	-	27.96%	36.56%	26%	31.07%
RRA	12.50%	18.75%	17%	5%	12.24%	-	8.60%		5%	
LGA			3%	2%					1%	4.85%
Others (SA and LHA)			2%	1%	0.34% (from LRA)			3.23% (from RIPA)		2.91%

A novel and distinctive aspect of the present study was the identification and classification of variant aortic origin IPAs. These aortic variant IPAs have not been systematically reported in the literature before. In the proposed classification, the IPAs arising at or above the level of the celiac trunk were categorized as Type I (normal), while those arising below the celiac trunk were termed Type II (displaced aortic origin).

The displaced IPAs (Type II) were further subclassified based on the extent of caudal descent in relation to the ventral branches of the abdominal aorta. Type II-1° displacement refers to arteries that originated below the level of the celiac trunk but above the SMA. Type II-2° displacement describes those arising precisely at the level of the SMA. Lastly, Type II-3° displacement encompassed arteries that originated below the SMA, typically in proximity to the renal artery. The distribution of these variants is detailed in Table 4.

**Table 4:** Variant aortic origin IPAs (displaced IPAs and degree of displacement) CA, celiac artery; IPA, inferior phrenic artery; SMA, superior mesenteric artery

Classification pattern of displaced IPAs	Level of origin	No. of arteries (n = 18)	Overall prevalence of displaced arteries
Type II: IPA origin below the origin of the CA			
(a) First-degree displacement	(a) Below the level of the celiac trunk between CA and SMA	10	4.93%
(b) Second-degree displacement	(b) Origin at the level of SMA	2	0.98%
(c) Third-degree displacement	(c) Below SMA	6	2.96%

All IPAs with non-aortic origins (arising from arteries such as celiac, left gastric, renal, splenic, or hepatic arteries) were categorized as Type III arteries. Thus, the proposed classification into Type I (normal aortic origin IPAs), Type II (displaced aortic origin variant IPAs), and Type III (non-aortic variant IPAs) provides a structured framework for describing IPA variations, with potential relevance for surgical, radiological, and oncological procedures.

Limitations

The present study had certain limitations. Firstly, the retrospective design may have introduced selection bias and limited control over potential confounding variables. The sample size, although comparable to similar CT-based anatomical studies, was modest (100 subjects) and may not capture the full spectrum of anatomical variations in the general population. Additionally, this was a single-center study, which may limit the generalizability of the findings. While the study primarily focused on the origin and pattern of IPAs, the complete anatomical course, branching details, and collateral connections of these arteries were not extensively assessed. Furthermore, the interpretation of CT angiograms was conducted without formal inter-observer variability assessment, and observer blinding was not implemented, which may impact the objectivity and reproducibility of findings. Lastly, no formal statistical sample size calculation or advanced statistical analysis was undertaken, as the study primarily aimed at descriptive anatomical categorization. Future multicentric studies with larger sample sizes, inclusion of inter-observer reliability, and correlation with surgical or cadaveric findings are recommended to strengthen and validate these observations.

## Conclusions

In the present study, the majority of IPAs originated from the abdominal aorta, with a total variation prevalence of 44.34% when right and left arteries were considered individually. A notably higher incidence of anomalous LIPAs arising from the left gastric artery, splenic artery, and left hepatic artery was observed compared to previous reports. The study uniquely proposed a novel classification system dividing IPAs into three types: Type I (normal aortic origin), Type II (displaced aortic origin), and Type III (non-aortic origin), with further subclassification of Type II into degrees of caudal displacement relative to the celiac trunk, SMA, and renal artery. To our knowledge, the description of displaced aortic origins of IPAs has not been previously reported. Comprehensive anatomical understanding of these different types of variants is crucial for improving surgical precision, enhancing interventional radiological techniques, and preventing inadvertent vascular complications, especially in hepatobiliary and upper abdominal surgeries performed under restricted operative fields.
